# Access to high-impact mutations constrains the evolution of antibiotic resistance in soft agar

**DOI:** 10.1038/s41598-018-34911-9

**Published:** 2018-11-19

**Authors:** Nour Ghaddar, Mona Hashemidahaj, Brandon L. Findlay

**Affiliations:** 1 Department of Chemistry and Biochemistry, Concordia University, Montreal, Québec Canada; 2 Present Address: Lady Davis Institute for Medical Research, McGill University, Montreal, Québec Canada

## Abstract

Despite widespread resistance to many important antibiotics, the factors that govern the emergence and prevalence of antibiotic-resistant bacteria are still unclear. When exposed to antibiotic gradients in soft agar plates measuring as little as 1.25 × 11 cm we found that *Escherichia coli* rapidly became resistant to representatives from every class of antibiotics active against Gram-negative bacteria. Evolution kinetics were independent of the frequency of spontaneous mutations that confer antibiotic resistance or antibiotic dose-response curves, and were only loosely correlated to maximal antibiotic concentrations. Instead, rapid evolution required unrealized mutations that could markedly decrease antibiotic susceptibility. When bacteria could not evolve through these “high-impact” mutations, populations frequently bottlenecked, reducing the number of cells from which mutants could arise and prolonging evolution times. This effect was independent of the antibiotic’s mechanism of action, and may affect the evolution of antibiotic resistance in clinical settings.

## Introduction

Antibiotic resistance is one of the greatest threats to modern medicine. Antibiotic-resistant organisms are thought to cause over 2,000,000 infections and 23,000 deaths in the United States each year^1^. Historically, preclinical assessment of new antibiotics has focused on measuring the rate with which resistance-conferring mutations occur *in vitro*^2^. Compounds with low or non-existent spontaneous resistance mutation rates are considered insensitive to the evolution of resistance *in vivo*, to the extent that some may theoretically be “evolution-proof”^3,4^. Unfortunately, clinical outcomes are less encouraging. The rate of spontaneous resistance development for clinical antibiotics span from 10^−5^ to 10^−11^ resistant mutations per cell per generation, and aren’t linked to the prevalence of resistance^2^. Rapid evolution of resistance during therapy also isn’t correlated to spontaneous resistance mutation rates; the leucyl-tRNA synthetase inhibitor GSK2251052/AN3365 recently failed a phase II clinical trial after rapid evolution of drug resistance *in vivo*, despite a mutation rate of 1.4 × 10^−7 ^^5,6^.

The recent development of laboratory evolution chambers has enabled directed evolution of bacteria without labour-intensive sub-culturing, allowing researchers to map the genotypic pathways to antibiotic resistance following growth in etched silicon wafers, moribodostats, and MEGA plates^7–9^. The latter has also revealed exciting evolution dynamics: bacteria growing in desk-sized MEGA plates encounter wedges of geometrically-increasing antibiotic concentrations up to 20,000x the initial MIC. In this environment, resistant mutants leave behind their susceptible brethren, providing an excellent example of how competition for nutrients can drive the evolution of antibiotic resistance^9^.

Unfortunately, operating these devices requires a combination of specialized expertise and custom-built equipment, limiting their throughput and general utility. To make the evolution of bacteria *in vitro* more accessible we have designed a compact system based on Soft Agar Gradient Evolution (SAGE). SAGE plates are built from standard petri dishes or similar labware, and can be run in high-throughput without ongoing operator involvement. We validated this system by generating mutants of *Escherichia coli (E*. *coli)* individually resistant to twelve antibiotics or antibiotic mixtures, covering every major antibiotic class active against Gram-negative bacteria. We then investigated the kinetics of *in vitro* evolution, using the fine control over selective pressure provided by the SAGE system to determine the key factors governing the rate of antibiotic resistance. We found that successful strains often exhibited mutation rates distinct from their progenitors, and that mutation supply rates didn’t constrain evolution rates in even small bacterial populations. Instead, evolution rates appeared to be constrained by the limited availability of potential high-impact mutations, unrealized changes to the genome that could markedly increase antibiotic resistance.

## Results

### Evolution chamber design

**Figure 1:**
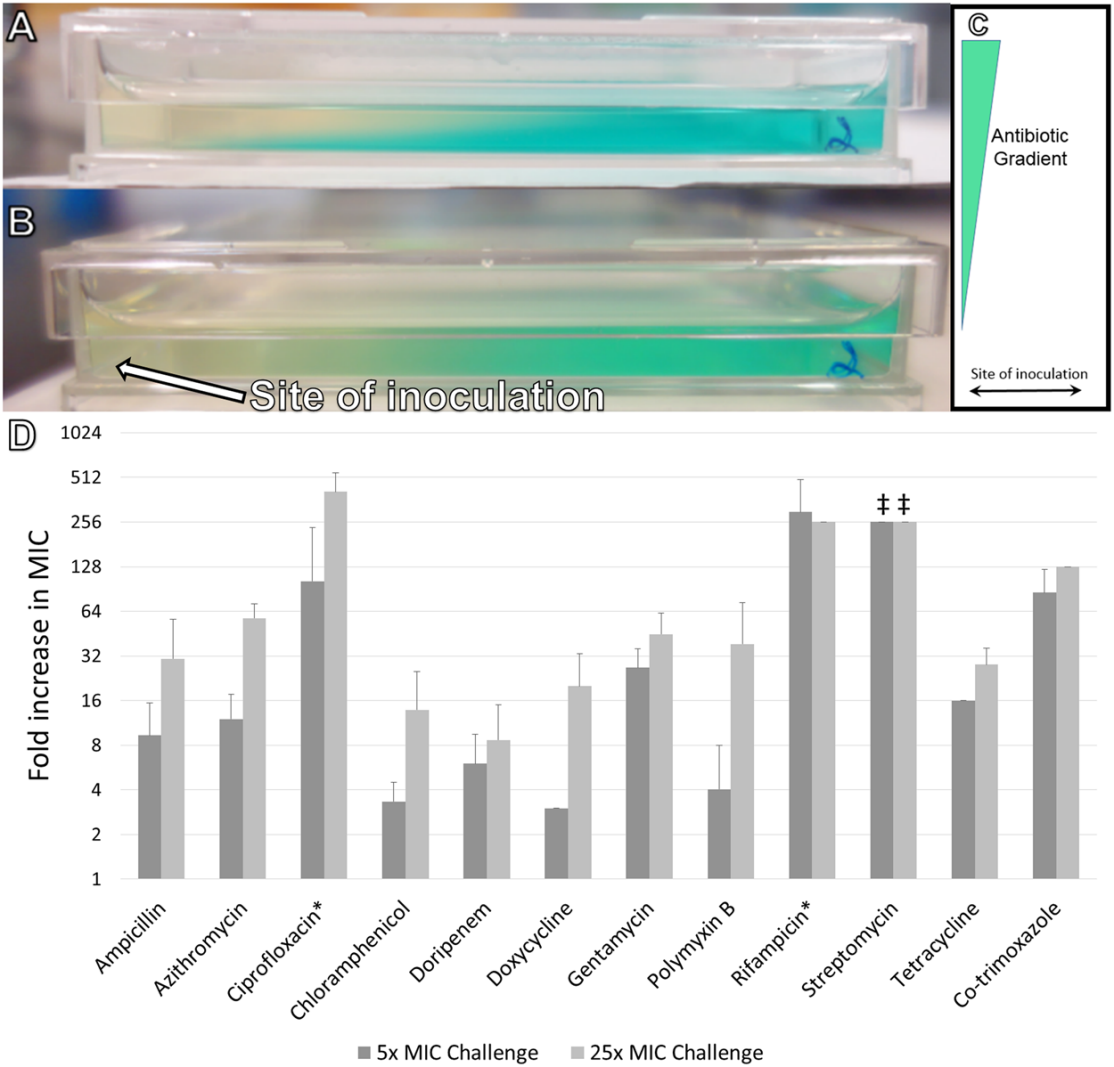
Evolution of antibiotic resistance. (**A**) Immediately after pouring the agar layers form two distinct wedges, as shown with the dye malachite green as a visual indicator. (**B**) After 16 hr of incubation the two layers have intermixed to create a smooth gradient of the dye. (**C**) A top-down schematic, showing the site of inoculation and chemical gradient. (**D**) Changes in minimum inhibitory concentration following antibiotic exposure, minimum of three biological replicates. Speed-selected *E*. *coli* MG1655 cells were passed through SAGE plates containing the listed antibiotics at approximately 5x the initial MIC value. Those which evolved resistance were then passed through a separate plate containing 25x the initial MIC. Individual tests are shown in Table S1. Lineages derived from the first passage were named e.g. Poly B 1, Poly B 2, etc., and lineages built by passing evolved strains through the 25x plates were correspondingly named Poly B 1-1, Poly B 1–2, etc. *Bacteria were exposed to plates containing 10x and 50x the initial MIC of ciprofloxacin, and 20x and 100x the initial MIC of rifampicin, respectively. ^‡^Resistance was greater than 256x the initial MIC. Cells were uniformly resistant to 8192 mg/L of streptomycin, and poor solubility limited testing at higher drug concentrations.

Hard agar gradient plates have been used since at least 1952 for antibiotic susceptibility testing^10^. They are created by sequentially pouring agar wedges of differing composition and allowing diffusion to equalize concentrations across the layers. By controlling the quantity of agar and the slope of the plate, it’s possible to create smooth gradients of arbitrary composition^10,11^. We found that these gradients are similarly stable at soft agar concentrations (0.2–0.75% agar w/v, Fig. 1), that allow bacteria to swim throughout the plate^12^. Bacteria inoculated into plates with antibiotic gradients grow until they encounter limiting concentrations of the antibiotic, at which point resistant mutants are selected for.

### Movement through soft agar

To select for efficient chemotaxis, *E*. *coli* MG1655, BW25113 *ΔmutL* and BW25113 *ΔmutS* were passed three times through 0.25% agar plates, at which point they were able to traverse the plate within 20 hours of incubation at 37 °C. Adding a thin layer of mineral oil both reduced syneresis and decreased the experiment times to approximately 4 hr (Figs S1–3). Bacteria on oil-containing plates had lower densities, with growth restricted to the upper millimetre of the gel (Fig. S3). Increasing the strength of the agar decreased bacterial movement in accordance with previous work on the movement of *E*. *coli* in soft agar^12^, while decreasing the agar load led to gels that were too fragile to handle efficiently.

Using the antibiotic ciprofloxacin at 75 μg/L (~5x the initial bacterial minimum inhibitory concentration (MIC)), we then screened for the evolution of antibiotic resistance in SAGE plates. Circular petri dishes, square petri dishes, machined polycarbonate plates of varying widths, and rectangular 4-well nunclon-treated plates all gave similar results (Fig. S2), with resistant cells emerging after 24 hr of incubation at 37 °C. Due to the similarities in outcomes all further evolution studies were performed with 4-well nunclon-treated plates.

Time-lapse microscopy revealed that *E*. *coli* moved smoothly through the agar medium (Fig. S4), propagating in a wave of high density cells^9,12^. Entering agar free of other strains, mutants were able to quickly establish a buffer of discarded cells, blocking competition from faster growing but more antibiotic-susceptible cells. This is similar to what was observed around the step gradients of MEGA plates^9^, and greatly reduced the impact of loss-of-fitness mutations, provided that these mutations also reduce antibiotic susceptibility.

### Evolution of antibiotic resistance

When challenged with a variety of antibiotics at a minimum of 5x the MIC, antibiotic resistance emerged in the three *E*. *coli* strains under investigation: speed-selected variants of *E*. *coli* MG1655 and two strains with elevated mutation rates due to defects in their mismatch repair systems; *E*. *coli* BW25113 Δ*mutS* and Δ*mutL*^13^. The magnitude of resistance varied by antibiotic, from 4x to 512x the initial MIC (Fig. 1D, Tables S1–3. Lower levels of resistance could in some cases be further increased by exposing mutants to a second SAGE plate containing 25x the original MIC.

Resistance evolved against every antibiotic tested as well as co-trimoxazole, a 1:19 mixture of trimethoprim and sulfamethoxazole. This included representatives from every major class of antibiotics, as well as antibiotics that don’t readily evolve resistance in other *in vitro* systems. E.g. resistance to both doxycycline and chloramphenicol increased 32-fold following passage through their respective SAGE plates, versus a 10-fold increase in doxycycline resistance after twenty-five days of growth in a morbidostat (compared to a 870-fold increase in chloramphenicol resistance over the same time period)^7^. Similarly, the ease with which we developed resistance to drugs like ampicillin, doripenem, and polymyxin B is at odds with antibiotic resistance in the clinic, which often takes years to gain prevalence in bacterial pathogens^14^. Clinical resistance is also often linked to the transfer of plasmid-encoded inactivating proteins, which were absent in this study^15^. For example, resistance to beta-lactams in *E*. *coli* is largely due to the production of beta-lactamases, but we were able to readily obtain mutants resistant to ampicillin and doripenem despite the fact that *E*. *coli* MG1655 lacks an inducible beta-lactamase (though a copy of *ampC* is expressed at low levels)^15–17^.

Both MG1655 and the mutator strains developed resistance at similar levels (Fig. 1D, Tables S1–3). Counter to a theoretical model comparing evolution under uniform and increasing drug concentrations^18^, the bacterial strains rapidly evolved resistance to both ciprofloxacin and streptomycin, despite the different fitness effects of their resistance-conferring mutations. This may be because the strong founder effect of the system limits competition between naïve and resistant strains, as detailed above.

### Genetic analysis

Amplification and sequencing of known proto-resistance genes identified several previously reported mutations in strains resistant to streptomycin, ciprofloxacin and trimethoprim (Tables S4 and S5). In particular, all four streptomycin-resistant lineages contained a K42R mutation in RpsL, which has been previously reported to confer near-immunity to streptomycin^19,20^. To evaluate broader genetic alterations we sequenced the genome of the progenitor strain of *E*. *coli* MG1655 and lineages resistant to ampicillin, gentamicin, or polymyxin B (Tables 1 and S6). The ancestral strain was found to have 78 mutations distinct from that of the previously published genome for *E*. *coli* MG1655^21^. Some of these were likely acquired prior to this work, but there was an interesting V42A mutation in LrhA, a transcriptional repressor of bacterial motility. This mutation has not been previously reported, but mutants lacking *lrhA* show enhanced chemotaxis^22^. It’s possible that the chemotaxis screen conducted at the start of this work selected for strains with deficient or altered LrhA function.

**Table 1:** Mutations identified through whole genome sequencing^a^.

Antibiotic^b^	Resistance-linked mutations	Mutations with unknown effect
Ampicillin	AcrB F628L	Aas D649G
	AcrR G28R	ElfG D140fs^c^
	AcrR C205T	WcaJ M375fs^c^
	EnvZ T250M	
	FtsI V545I^c^	
	MarR L46F, C108T	
Gentamicin	CpxA A97T	HofM T133A
	DinF W455R	MalF Q115*
	FusA A592V, A608V	RfaH D106D
	SbmA D33fs	
	YbhS L216P	
Polymyxin B	AcrB G861E	Aas D649G
	ArnC A192V	AsmA R86C, E524fs
	BamA Q441R, D447G	HofM T133A
	BasR G53E	MalF Q115*
	BasS L14P	MalI T207A
	Gmd F21L, T27C	RfaH D106D
	LptD G701R	YfiM A106H
	LpxC G106S	

^a^For a full list see Table S7.

^b^Strains were collected following passage through the same 5x SAGE plate but different 25x plates.

^c^Mutation found in both lineages.

Correcting for mutations in the ancestral strain, we found between 29 and 178 single nucleotide polymorphisms in the evolved strains (Table 1; a full list of mutations can be found in Table S6). In general, resistance appeared to be conferred not by any single genetic change but rather by the additive effects of a small series of mutations. For example, both ampicillin-resistant mutants contained a V545I mutation in penicillin-binding protein 3, FtsI. This mutation is linked to a roughly 3-fold increase in beta-lactam resistance in *Salmonella* spp.^23^, but the MIC of ampicillin rose 64-fold following passage through SAGE plates. The further increase in MIC appears to be related to missense mutations in the AcrAB efflux pump and its regulators, AcrR and MarR. Mutations in AcrR and MarR have been previously linked to a 4-fold increase in ampicillin resistance, by increasing expression of AcrAB and thereby reducing drug uptake^24^. Similarly, while the polymyxin B-resistant lineages contained a number of mutations in proteins involved in LPS biosynthesis or modification, they also contained mutations not previously linked to polymyxin B resistance, including in MalF, YfiM, and HofM. These proteins are found in the cell envelope, and may be involved in movement of polymyxin B across the membrane. Gentamicin and polymyxin B both traverse the outer membrane through self-promoted uptake^25^ and gentamicin-resistant lineages shared mutations in MalF and HofM (Table 1). Strains of *E*. *coli* deficient in *malF* and *hofM* were acquired from the Keio collection, but showed no appreciable difference in their MIC values to polymyxin B. The change in MIC conferred by these mutations may be lower than our limit of detection (one doubling of antibiotic concentration).

### Mutation rate and population size

While no mutations in mismatch repair were observed during whole genome sequencing, we were concerned that passage through SAGE plates might select for mutators: strains with constitutively high mutation rates. We therefore determined the mutation rate of several strains before and after passage through SAGE plates, using the frequency of spontaneous resistance to rifampicin as a proxy measurement (Table 2)^26,27^. Mutation rates for all three progenitor strains (MG1655, BW25113 *ΔmutL* and BW25113 *ΔmutS*) were altered during the speed selection process, elevating the mutation rate of MG1655 while reducing the mutation rates of BW25113 *ΔmutL* and BW25113 *ΔmutS*. Subsequent passage through SAGE plates containing polymyxin B or ampicillin further altered the frequency of spontaneous resistance to rifampicin in MG1655, leading to lineages with rates both above (MG1655 Poly B 1 and 2) and below (MG1655 Poly B 1-1 and 1–2) that of the speed selected progenitor.

**Table 2:** Frequency of spontaneous rifampicin resistance before and after antibiotic exposure.

Lineage	*f* (*×10*^8^)^b^	μ (×10^8^)
MG1655	4.0 ± 2.1	1.1
MG1655 Post speed selection^a^	64.6 ± 9.8	12.8
*E*. *coli* BW25113 ΔmutL	188 ± 4.3	31.1
*E*. *coli* BW25113 ΔmutL Post speed selection^a^	19.2 ± 12.8	4.3
*E*. *coli* BW25113 ΔmutS	650 ± 134.5	82.9
*E*. *coli* BW25113 ΔmutS Post speed selection^a^	45.3 ± 4.0	7.8
Ampicillin 1-1	57.3 ± 27.7	18.3
Polymyxin B 1	312 ± 90	66.8
Polymyxin B 1-1	17.1 ± 6.2	6.4
Polymyxin B 1-2	15.6 ± 2.7	5.8
Polymyxin B 2	71.6 ± 13.4	19.9

^a^After selection for efficient swimmers, prior to antibiotic exposure. ^b^Average of three technical replicates.

To gain a clearer picture of the mutation supply rate, we estimated the population size in SAGE plates. Representative samples from the growing front and stationary lawn of antibiotic-free SAGE plates were excised, and the cell counts in each sample were determined via qPCR using universal 16 S primers and a dilution standard of genomic *E*. *coli* DNA. qPCR is often used to measure populations of environmental bacteria^28^ and was favoured here as the bacterial cells of interest were embedded within an agar matrix. Relating our results back to the width of the excisions (~0.75 cm) and depth of the plate (0.45 cm), we found that a growing front contains approximately 1.31 × 10^7^ cells/cm (s = 1.0 × 10^6^), and that the density of cells behind this front is approximately 5.98 × 10^7^ cells/cm^2^ (s = 3.2 × 10^7^). Only cells in the front are under selection, and so the maximal population under selection is 3.93 × 10^7^ cells (s = 3.0 × 10^6^). This value is likely to be an overestimate, both due to the likely presence of a small number of dead and dormant cells, and because during antibiotic selection the front often fragments into distinct lineages, creating a number of smaller sub-populations (see Fig. 3; below).

### Factors governing the evolution of antibiotic resistance

#### Maximal antibiotic concentration and slope

The time required for bacteria to evolve antibiotic resistance was consistent across replicate experiments for each antibiotic investigated, but varied between drugs. To determine the factors that governed the evolution of resistance we began to vary the slope and concentration of antibiotic that bacteria were exposed to.

**Figure 2:**
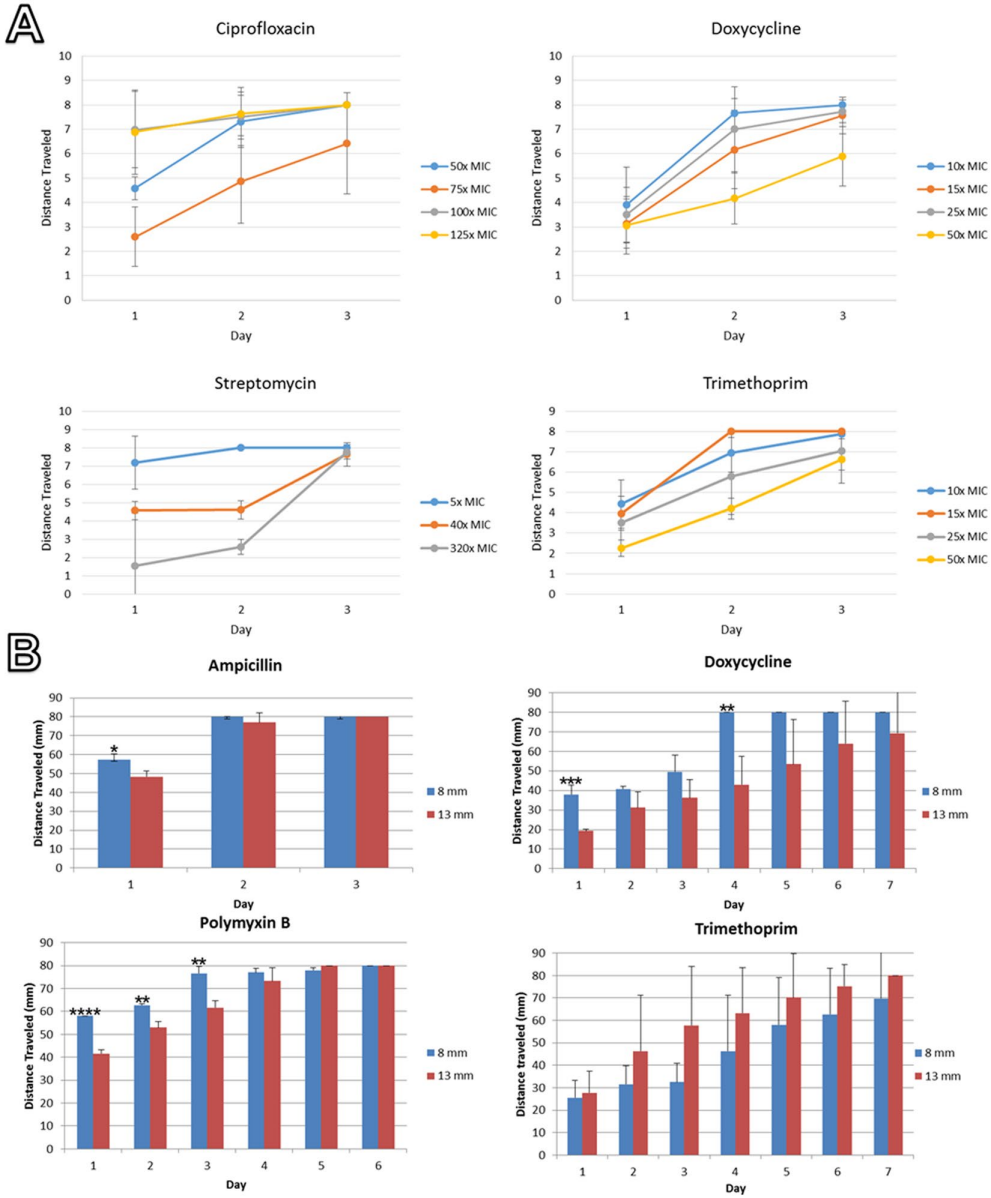
Passage through SAGE plates as a function of (**A**) antibiotic concentration and (**B**) slope. Averages of three biological replicates are shown. Antibiotic concentrations are given as the maximal concentration in the plate, as a function of MIC. The slope in (**A**) was 8 mm. (**B**) The likelihood that the observed differences in movement were due to chance was calculated via a two-tailed unequal variance Student’s T test.*P value < 0.05, **P value < 0.01, ***P value < 0001, ****P value < 0.00001.

Increasing the maximal concentration of streptomycin in the plates from 5x MIC to 40x MIC and 320x MIC decreased growth across the plate during the first two days of incubation, but had no statistically significant effect on growth at 72 hours. (Fig. 2A). Other antibiotics showed a similar pattern, though with ciprofloxacin increasing the maximal drug concentration from 75x MIC to 125x MIC actually accelerated growth. This may be due to the mutagenic effect of subinhibitory ciprofloxacin concentrations, which could potentially increase the effective mutant supply rate^29^. Of the antibiotics tested, only doxycycline showed a statistically significant difference in growth at 72 hr (10x MIC vs 50x MIC, s = 0.04, two-tailed, unequal variance Student’s t-test).

Increasing the slope of the antibiotic gradients from 8 mm to 12.5 mm also gave mixed results (Fig. 2B). No significant difference in movement rate was observed with plates containing trimethoprim, but differences of varying magnitude were observed in SAGE plates laced with ampicillin, doxycycline, and polymyxin B. The effect was pronounced with the latter two antibiotics at 25x the initial MIC, with much slower movement across the 13 mm plates.

#### Antibiotic dose-response

In SAGE plates selective pressure is restricted to a narrow, high-density band of growing cells (Fig. S4; see above). Varying the maximal antibiotic concentration or slope may reduce the width of this band, constricting the population under selection and causing the prolonged experiment times we observed^30^. As changing the antibiotic concentration could also alter the mutations required for resistance we investigated the correlation between evolution time and band width through the antibiotic dose-response curve^31^. Compounds with steep dose-response curves will rapidly lower bacterial growth rates as the concentration of drug increases, reducing the width of the growing band. The antibiotic dose-response curve is also closely linked to the mutant selective window in other evolution systems, and is thought to contribute to the slow evolution rate of resistance against antimicrobial peptides like polymyxin B^30,32^.

However, we found no correlation between the dose-response curves of antibiotics and evolution rates (Table 3). Populations of fast-swimming *E*. *coli* MG1655 exposed to both doxycycline and polymyxin B required more time to traverse SAGE plates than populations exposed to ciprofloxacin or trimethoprim, though doxycycline had the shallowest measured dose-response curve and polymyxin B the steepest.

**Table 3:** Antibiotic dose-response and evolution rates.

Antibiotic	N value	Median Time to Completion^a^	
		15 × MIC	25 × MIC
Ciprofloxacin	3.2	2	2
Doxycycline	1.3	3	5
Polymyxin B	32.2	3	6
Trimethoprim	3.3	2	2

^a^Median of three biological replicates. All values in days. N values for *E. coli* MG1655 were calculated after speed selection. Large N values correspond to steep dose response curves.

### The impact of resistance-conferring mutations determines evolution rates

**Figure 3:**
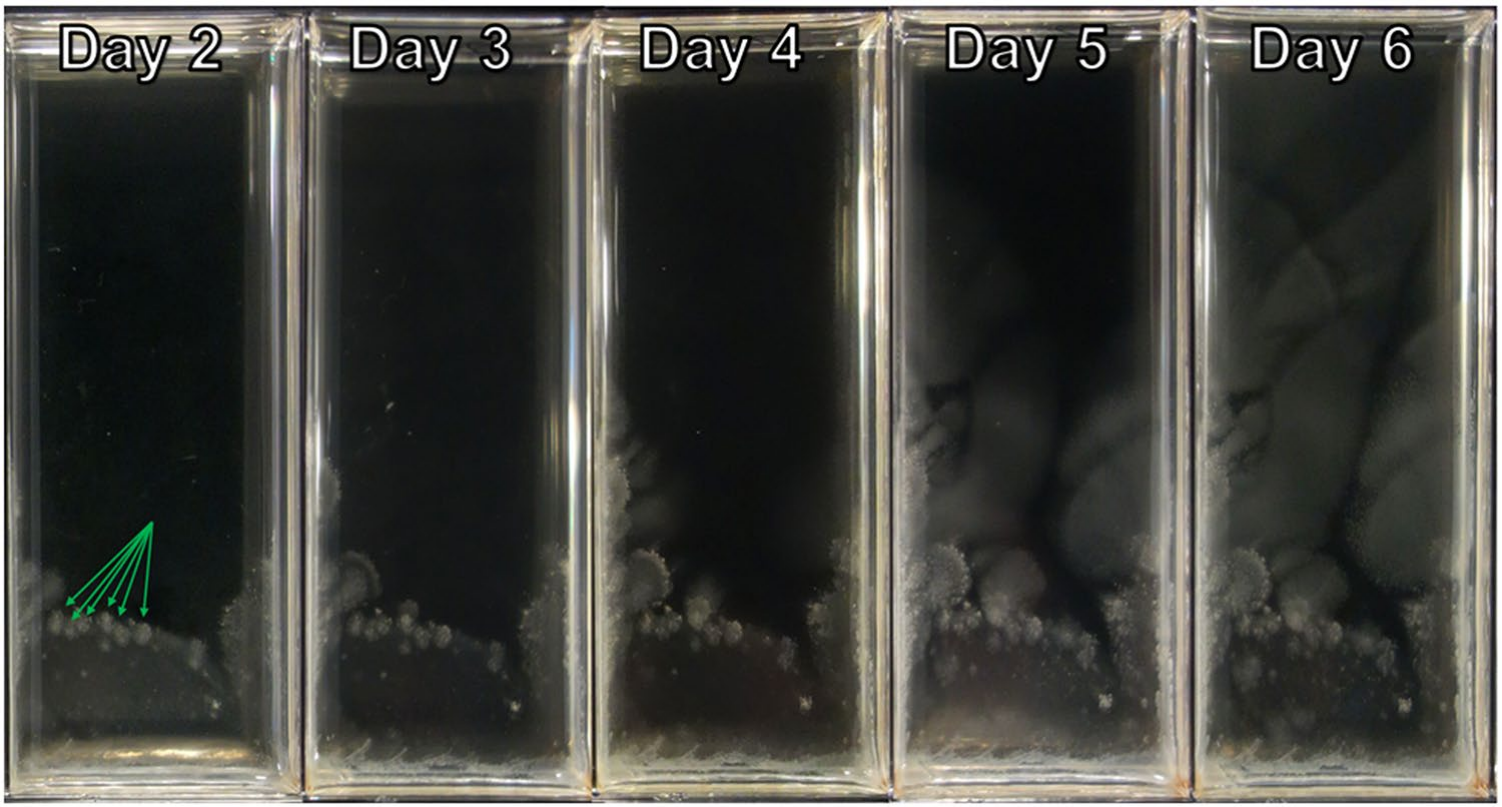
Visual observation of resistance to doxycycline. SAGE plates were incubated at 37 °C for seven days. Shown here is one representative lane, containing up to 25x the initial MIC of doxycycline at a slope of 12.5 mm. Nodules of resistant cells observed on day 2 are marked by arrows. With high concentrations of doxycycline, many of these mutations conferred only a small increase in MIC, leading to small populations of moderately resistant bacteria. Large bacterial populations did not reform until day 5.

At elevated antibiotic concentrations the dynamics of growth across SAGE plates changed. While bacteria still began by growing in a band of high-density cells, movement at growth-limiting concentrations of antibiotic was slow enough that individual mutation events were visible (Figs 3 and S4). Mutants first appeared as pinpricks of high density cells, growing over time into cones as cells radiated out from their point of origin. In trimethoprim plates these cones quickly coalesced into new bands of high-density cells, which moved across the plate until the concentration of antibiotic was again growth-limiting (Fig. S4).

Mutations were also observed in doxycycline plates, but the change in MIC provided by these mutations was much smaller. As a result, the majority of mutants didn’t form large cones, but instead stopped growing near the point of mutation, leaving behind small nodules of high-density cells (Fig. 3, arrows). Over several days the bacteria in these nodules mutated further, increasing their resistance and allowing further movement across the plate. Increasing the plate’s slope significantly compressed the nodules, reducing the population that could give rise to highly-resistant mutants. This in turn increased the amount of time required for doxycycline resistance to evolve.

Thus, the rate of evolution in SAGE plates is set by the change in antibiotic susceptibility conferred by unrealized mutations. Latent “high-impact” mutations allow strains to re-establish large populations following antibiotic challenge, ensuring that if the concentration of antibiotic once again becomes growth limiting there is a large population from which further mutants can arise. When resistance is conferred by low-impact mutations, the bacteria become trapped in population bottlenecks, reducing the likelihood of further mutations.

With this new framework, we can explain the rapid evolution of bacteria exposed to high levels of ciprofloxacin, trimethoprim, and streptomycin. Resistance to each of these antibiotics is known to occur from a small number of high-impact mutations^19,33,34^, several of which were observed in strains following passage through SAGE plates (Table S5). In principle, any antibiotic whose activity can be sharply curtailed by a small number of high-impact mutations will be quickly rendered ineffective by SAGE, regardless of the spontaneous resistance mutation rate or maximal antibiotic concentration.

### Population mutation rates appear to vary within SAGE plates

The population bottlenecks that antibiotics create within SAGE plates may be partially alleviated by transiently increasing the mutation supply rate by rapid cell turnover^35^. Antibiotic stress is also known to trigger transiently elevated mutation rates through the SOS response, the stringent response, and the production of reactive oxygen species^36–38^. However, our work suggests that in SAGE plates these bacteria may also improve their mutation supply rate through the transient evolution of strains with high background mutation rates.

High mutation rates are known to help populations more easily develop antibiotic resistance^37^, and mutator strains are more prevalent in populations of bacterial pathogens^39,40^. However, we found that mutators were uncommon following exposure of speed selected *E*. *coli* MG1655 to gradients of polymyxin B, and that simple passage of *E*. *coli* BW25114 *ΔmutS* through antibiotic-free agar was sufficient to drop the observed mutation rate 14-fold, bringing it close to values recorded for the other strains investigated (Table 2, see above). High mutation rates can reduce fitness in the absence of stress^40,41^ and we hypothesize that this is leading to counter-selection later in the plate, when the bulk of the population is antibiotic resistant.

Similar behaviour has been recently observed in *E*. *coli* populations exposed to ethanol stress, where population mutation rates frequently rose and fell in response to increasing ethanol concentrations^42^. If this process is occurring in the SAGE system it could explain why we observed relatively small populations of both wildtype progenitor strains and progenitors with mutator phenotypes rapidly developing resistance to antibiotics with low spontaneous resistance mutation rates.

### Other environments

Extending these results to bacterial evolution in other systems, access to high-impact mutations may broadly constrain the *in vitro* evolution of antibiotic resistance. Resistant mutants are often less fit, and in liquid environments like that of morbidostats don’t form a significant fraction of the population until the antibiotic significantly inhibits susceptible strains^43^. When bacteria evolve resistance through a series of low-impact mutations these *in vitro* systems may be unable to distinguish between the susceptibility of naïve and resistant strains. This may be the case with doxycycline resistance, which generally doesn’t exceed 10x the initial MIC following either serial passage through liquid media or growth in a morbidostat^7,44^. The low-level mutants we observed in SAGE plates may occur in these systems, but the concentration of doxycycline needed to inhibit the growth of susceptible strains will likely inhibit the mutants as well, preventing them from forming the majority of the population or evolving elevated levels of resistance.

## Discussion

Using antibiotic gradients in soft agar we evolved mutants of *E*. *coli* resistant to representatives of every major class of antibiotic active against Gram-negative bacteria. Our findings show that in soft agar gradients evolution kinetics are constrained by the potential for high-impact mutations. When bacteria develop resistance through high-impact mutations, as with streptomycin resistance, bacteria quickly recover from population bottlenecks following selection, easing passage through future selective barriers. In contrast, factors that have been previously linked to the rate of resistance development, such as the maximal antibiotic concentration, spontaneous resistance mutation frequency, and antibiotic dose-response/mutant selection window had little influence on evolutionary success. Their limited impact in the SAGE plates appears to be in part due to the transient evolution of strains with elevated mutation rates, which increases the mutation supply rate and allows for the generation of resistant mutants from even small bacterial populations. In the absence of antibiotic stress these hypermutators appear to be rapidly replaced by strains with lower mutation rates.

Natural environments are unlikely to contain semi-solid agar gels, but will likely contain antibiotic gradients, potentially allowing for evolutionary dynamics similar to what we have observed. In particular, the human body readily creates drug gradients during both antibiotic therapy and chemotherapy, due to limited penetration into various tissues^45–47^. If these gradients accelerate the evolution of resistance *in vivo*, this likely explains why our current methods of evaluating potential new antibiotics are poorly correlated to clinical outcomes^2^. To reduce the emergence of resistance, antibiotic candidates should instead be evaluated by their potential to be nullified by a small number high-impact mutation. Those which lack such convenient resistance pathways will be less susceptible to the evolution of resistance.

## Experimental Procedures

### Soft agar gradient evolution plates

Sterile 4-well nunclon treated culture dishes were purchased from Thermo Scientific (cat. 167063), and used for the majority of evolution experiments. The hydrophilic surface treatment was not critical for SAGE experiments, but led to more resilient gels due to increased interactions between gel and plate.

### Strains

*E*. *coli* MG1655 was a generous gift from Éric Déziel, INRS, Canada. *E*. *coli* BW25113 ∆mutS and ∆mutL were purchased from the Coli Genetic Stock Center (CGSC) and are part of the Keio Collection^13^.

### Soft agar gradient evolution experiments

Molten 0.25% cation-adjusted Mueller-Hinton agar (MHA) was poured into 4-well plates that were raised on one side 3 mm, 8 mm, or 12.5 mm. Convenient lifters were made from P200 pipette tips, P1000 pipette tips, or a 1/2” role of labeling tape, respectively. Agar was added to half the height of the well on the lower side (0.45 cm), then left to gel at room temperature for 20 minutes. The supports were then removed and a second agar solution was added to an even depth. Plates were incubated overnight at room temperature to allow diffusion between the two layers.

To initiate experiments up to 50 µL of an overnight bacterial culture was inoculated in a line on the side of the well where the concentration of antibiotic was lowest. The wells were then covered with up to 5 mL of mineral oil to prevent desiccation and incubated at 37 °C.

After cells had grown throughout the plate mutants were harvested by sampling relevant regions of the plate via pipette. The soft agar extracted was then added to 5 mL of cation-adjusted Mueller-Hinton Broth (MHB) and incubated overnight at 37 °C. Cells were stored at −80 °C in glycerol or used for further experiments.

### Minimum inhibitory concentration measurements

Populations extracted from SAGE plates were assessed following standard microdilution procedures^48^. In brief, 50 µL of cation-adjusted MHB containing the antibiotic of interest was mixed 1:1 with fresh media containing approximately 1 × 10^6^ bacterial cells (verified against a freshly prepared McFarland 0.5 standard) to give a final cell density of 5 × 10^5^ CFU. Plates were then incubated at 37 °C for 16–20 hours. Wells lacking bacteria were used as negative controls, and wells with bacteria and without antibiotic were used as positive controls. The MIC was defined as the minimum concentration of antibiotic that results in no visible growth to the naked eye.

### Dose response curves and growth rate measurements

To measure the growth rate of *E*.*coli* in the presence of different concentrations of each antibiotic, bacteria were added to 96-well plates as described above. The plates were then sealed with parafilm and incubated at 30 °C in a Tecan Sunrise plate reader running Magellan V 7.1. Absorbance was measured at 595 nm every 30 seconds, with 15 seconds of shaking and two seconds of settle time prior to each measurement. Measurements were collected for 23 hours. The data was then exported to Excel. Measurements were zeroed and converted to their natural log. The steepest region of the growth curve was then determined, and a minimum of forty measurements were then fit to a linear curve. These dose response curves were then plotted against antibiotic concentration and fit to a Hill function via linear regression with the Excel Solver tool^31,49^:

$$g(c)=\frac{{g}_{0}}{1+{(\frac{c}{I{C}_{50}})}^{n}}.$$

where, g(c) is growth rate as a function of antibiotic concentration, c, g_0_ is the growth rate in the absence of antibiotic, and IC_50_ is the concentration of antibiotic where the growth rate was half g_0,_ n is the hill co-efficient, and corresponds to how quickly growth decreases as the antibiotic concentration increases.

### Mutation rates

To infer the mutation rate of evolved *E*. *coli* lineages we measured the frequency of spontaneous resistance to rifampicin^26^. In brief, each strain was grown in MHB media overnight. Cells were then diluted in fresh MHB and plated in triplicate onto either LB-agar or LB-agar with 100 µg/mL rifampicin. The plates were incubated for 16–20 hours at 37 °C, and the number of colonies on each was counted. The median frequency, *f*, of each strain was then calculated and used to determine the median mutation rate, μ, based on the formula:

$$\,{\rm{\mu }}={\rm{f}}/ln(N\mu )$$

where N is the total population, determined by counting cells grown on rifampicin-free agar^26,50^.

### Whole genome sequencing

Strains of interest were grown overnight in MHB, then genomic DNA was extracted using the EZ-10 Bacterial Genomic DNA miniprep kit according to manufacturer’s specifications. Samples were sequenced by Genome Quebec on an Illumina HiSeq. 4000 PE100. Sequence data was then assembled with the A5-miseq pipeline software. Snippy was used to compare the genome assemblies to the reference genome of *E*. *coli* MG1655, U00096.3 (https://github.com/tseemann/snippy).

### Population size calculations

SAGE plates free of antibiotic were inoculated with 50 μL of *E*. *coli* MG1655, then incubated at 37 °C for four hours. Agar blocks containing representative bands and bacterial lawns were then excised with a sterile spatula and heated at 98 °C for five minutes. Cells were mixed 1:10 with distilled, deionized water, heated at 98 °C for three more minutes, and then used as template for qPCR. In brief, 1 μL of template was mixed with 14.6 μL ddH_2_O, 4 μL 5X EvaGreen qPCR mix, 0.4 μL forward primer (0.1 μM, 16 S F27) and 0.4 μL reverse primer (0.1 μM, 16 S R1492)^51^. The thermal profile used was 15 min of polymerase activation at 95 °C followed by the PCR cycling stage with 40 cycles (95 °C for 45 sec, 55 °C for 30 sec, 72 °C for 1 min) and ending with a melting curve (95 °C for 15 sec, 55 °C for 15 sec, 95 °C for 15 sec). Results from a dilution series of genomic DNA were used to determine the cell copy numbers.

For cells extracted from the growing front, the reported number of cells was multiplied by 10 to account for the initial dilution, then standardized to the total volume of the excised gel band. This population was then divided by the width of the excised band (~0.75 cm) to give a cell count per cm of the growing front. Values corresponding to cells extracted from the lawn were multiplied by 10, divided 0.05 mL and multiplied by sqrt (1 cm^3^/0.45 cm) = 1.49 cm^2^ to adjust for the depth of the agar (0.45 cm). The total population of the growing front was estimated by multiplying the width of the lane (2.9 cm), the width of the growing front in the absence of antibiotic (0.3 cm) and the depth of the agar which contained cells (0.1 cm). Results are presented as the average of three independent replicates.

## Electronic supplementary material

Supplementary Data

S6

## Data Availability

Full MIC testing results and whole-genome sequencing data are included in the supplementary information files.
