# Supplementary material for: Access to high-impact mutations constrains the evolution of antibiotic resistance in soft agar
Source: Sci Rep. 2018 Nov 19;8:17023. doi: 10.1038/s41598-018-34911-9 (PMC6242871; doi:10.1038/s41598-018-34911-9)
Supplement: Supplementary file 1 — Supplementary Data [file 41598_2018_34911_MOESM1_ESM.docx]

**Access to high-impact mutations constrains the evolution of antibiotic resistance in soft agar**

Nour Ghaddar^1,#,¶^, Mona Hashemidahaj^1,¶^, Brandon L. Findlay^1,*^

Affiliations:

^1^ Department of Chemistry and Biochemistry, Concordia University, Montreal, Québec, Canada

^#^ Current address: Lady Davis Institute for Medical Research, McGill University, Montreal, Québec, Canada

^*^ Corresponding author.

Brandon L. Findlay, Email: brandon.findlay@concordia.ca

^¶^ These authors contributed equally.

**PCR amplification and sequencing**

Genes of interest were amplified from whole cells via colony-pick PCR. A geographically distinct colony was transferred from hard agar plate to 100 μL of sterile ddH_2_O. The sample was then heated to 95 ℃ for five minutes and used directly as the template for subsequent PCR reactions.

**Table S1.** Minimum Inhibitory Concentrations following evolution of speed selected *E. coli* M1655 via SAGE plates.

|  | Lineage | | | | | | | | | |
| --- | --- | --- | --- | --- | --- | --- | --- | --- | --- | --- |
|  | Naïve | 1 | 2 | 3 | 1-1 | 1-2 | 2-1 | 2-2 | 3-1 | 3-2 |
| Ampicillin | 8^a^ | 128 | 64 | 32 | 512 | 512 | 128 | 128 | 64 | 128 |
| Azithromycin | 4 | <16 | 64 | 32 | 128 | - | 256 | 256 | 256 | 256 |
| Ciprofloxacin | 0.0075 | 0.188 | 0.188 | 1.92 | 3.84 | 3.84 | - | 3.84 | 1.92 | 1.92 |
| Chloramphenicol | 16 | 64 | 64 | 32 | 512 | 256 | 256 | 256 | 16 | 32 |
| Doripenem | 0.06 | 0.48 | 0.48 | 0.12 | 0.96 | 0.96 | 0.48 | 0.48 | 0.12 | 0.12 |
| Doxycycline | 1 | 3 | <16 | 3 | 6 | - | 32 | 32 | 6 | 24 |
| Gentamycin | 1 | 32 | 32 | 16 | 32 | 64 | 64 | 32 | 32 | - |
| Polymyxin B | 0.5 | 4 | 2 | >0.0625 | 32 | 32 | - | 32 | >0.0625 | >0.0625 |
| Rifampicin | 8 | 1024 | 2048 | >4096 | 2048 | 2048 | 2048 | 2048 | - | - |
| Streptomycin | 32 | >8192 | >8192 | >8192 | >8192 | >8192 | >8192 | >8192 | >8192 | >8192 |
| Tetracycline | 0.5 | 8 | 8 | <0.5 | 16 | 8 | 16 | - | 16 | - |
| Trimethoprim/  Sulfamethoxazole | 0.0625/  1.1875 | 4/76 | 4/76 | 8/152 | 8/152 | 8/152 | 8/152 | 8/152 | 8/152 | - |

^a^ MIC. All values in mg/L.

**Table S2.** Minimum Inhibitory Concentrations following evolution of speed selected *E. coli* BW25113 Δ*mutL* via SAGE plates.

|  | Lineage | | | | | | |  |
| --- | --- | --- | --- | --- | --- | --- | --- | --- |
| Antibiotic | Naïve | 1 | 1-1 | 1-2 | 2 | 2-1 | 2-2 | |
| Ampicillin | 4^a^ | - | 512 | - | 64 | - | - | |
| Azithromycin | 16 | - | 512 | 512 | 128 | - | - | |
| Ciprofloxacin | 0.015 | 0.12 | 3.84 | 3.84 | 0.12 | 1.92 | 3.84 | |
| Chloramphenicol |  |  |  |  |  |  |  | |
| Doripenem | 0.12 | - | 0.48 | 0.96 | 0.48 | - | - | |
| Doxicycline | 16 | <16 | 64 | 64 | <16 | 64 | 32 | |
| Gentamycin | 2 | 32 | 64 | 64 | 64 | 32 | 64 | |
| Polymyxin B | 0.5 | 4 | 64 | 64 | 2 | 64 | 32 | |
| Rifampicin | 8 | 2048 | 2048 | 2048 | 2048 | 2048 | 4096 | |
| Streptomycin | 16 | >8192 | 8192 | 8192 | 8192 | >8192 | >8192 | |
| Tetracycline | 4 | - | 32 | 32 | 8 | - | - | |
| Trimethoprim  /Sulfa | 0.125  /2.375 | - | 16/304 | 8/152 | 8/152 | - | - | |

^a^ MIC. All values in mg/L.

**Table S3.** Minimum Inhibitory Concentrations following evolution of speed selected *E. coli* BW25113 Δ*mutS* via SAGE plates.

|  | Lineage | | | | | | |
| --- | --- | --- | --- | --- | --- | --- | --- |
| Antibiotic | Naïve | 1 | 1-1 | 1-2 | 2 | 2-1 | 2-2 |
| Ampicillin | - | - | - | - | - | - | - |
| Azithromycin | 32^a^ | 128 | 512 | 512 | - | - | - |
| Ciprofloxacin | - | - | - | - | - | - | - |
| Chloramphenicol | 8 | 16 | - | - | 16 | 128 | 64 |
| Doripenem | 0.06 | 0.48 | 0.96 | 0.96 | 0.48 | 0.96 | 0.96 |
| Doxicycline | - | 32 | 64 | 64 | 32 | 64 | 64 |
| Gentamycin | 1 | 16 | 32 | 32 | 32 | 32 | 32 |
| Polymyxin B | <0.25 | 8 | 32 | 8 | 1 | - | - |
| Rifampicin | 16 | 1024 | 2048 | 2048 | 1024 | 2048 | 2048 |
| Streptomycin | 16 | 8192 | 8192 | 8192 | 8192 | 8192 | 8192 |
| Tetracycline | 2 | 32 | 64 | 64 | 16 | 64 | 64 |
| Trimethoprim/  Sulfa | 0.125/  2.375 | <0.25/4.75 | 8/152 | 8/152 | <0.25/  4.75 | 4/76 | - |

^a^ MIC. All values in mg/L.

**Table S4.** Primers used for PCR amplification and sequencing.

| Amplified gene | Primers | Primer used for sequencing |
| --- | --- | --- |
| *rrl* | 1. FW: 5’- TGAAGCAACAAATGCCCTGC-3’ 2. Rev: 5’- ACTGGCGTCCACACTTCAAA -3’ | 2 |
| *gyrA* | 1. FW: 5’- CGACCTTGCGAGAGAAAT-3’ 2. Rev: 5’-GTTCCATCAGCCCTTCAA-3’ | 3 |
| *parC* | 1. FW: 5’-GTGCGTTGCCGTTTATTGGT-3’ 2. Rev: 5’-ACTGAACCAGTCCGTTCTC-3’ | 5 |
| *rpsL* | 1. FW: 5’- ACCATTAAGCCTTAGGACGCT-3’ 2. Rev: 5’-GCAAAAGCTAAAACCAGGAGCTA-3’ | 13 |
| *rrs* | 1. FW: 5’-TCAGGCGGTGAAACGGATAC-3’ 2. Rev: 5’-AACCACCTGAGCTACAAGCC-3’ 3. FW: 5’ –TGCCTGATGGAGGGGGATAA-3’ | 17 |
| *folP* | 1. FW: 5’ – GGACCTTAGCCATCCTCACG-3’ 2. Rev: 5’- TCCTTTGCCATCCTCACG- 3’ | 18 |

**Table S5.** Resistance-conferring mutations in *E. coli* identified by PCR and Sanger sequencing.

| **Antibiotic** | **Lineage^a^** | **Resistance-conferring Mutations^b^** |
| --- | --- | --- |
| Ciprofloxacin^c^ | 1 | GyrA S83L |
|  | 1-1 | GyrA S83L |
| Sulfamethoxazole/ | 1 | FolP P64S |
| Trimethoprim^d^ | 1-1 | FolP T62A |
| Streptomycin | 1 | RpsL K42R |
|  | 1-1 | RpsL K42R, *rrs* C941T |

^a^ Strains were collected following passage of *E. coli* MG1655 through SAGE plates containing a maximal concentration of 5x the MIC of the listed antibiotic (1). Mutated strains were then passed through a second plate containing 25x the listed antibiotic (1-1). Single colonies were picked for sequencing.

^b^ A full list of the genes and primers investigated can be found in Table S4.

^c^ Ciprofloxacin plates contained 10x and 50x the MIC.

^d^ 19:1 mixture.

**Supplementary Figures**

**
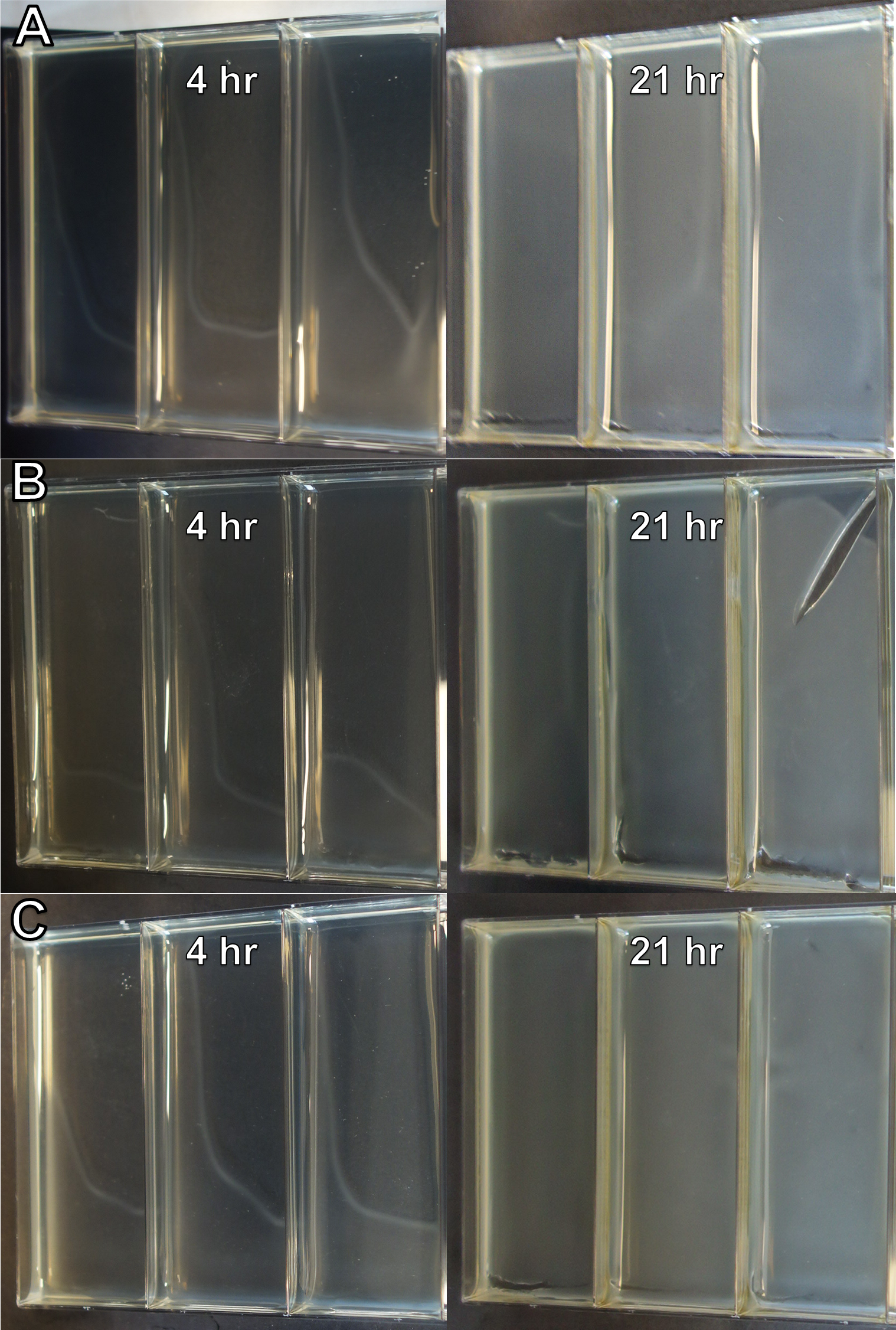
**

**Fig S1.** Growth of bacteria in soft agar. 50 uL of speed-selected A) *E. coli* MG1655, B) *E. coli* BW25113 *ΔmutL* and C) *E. coli* BW25113 *ΔmutS* were inoculated in triplicate into 0.25% Mueller Hinton agar (MHA). Plates were then incubated at 37 ℃. The three strains showed similar growth patterns.

**
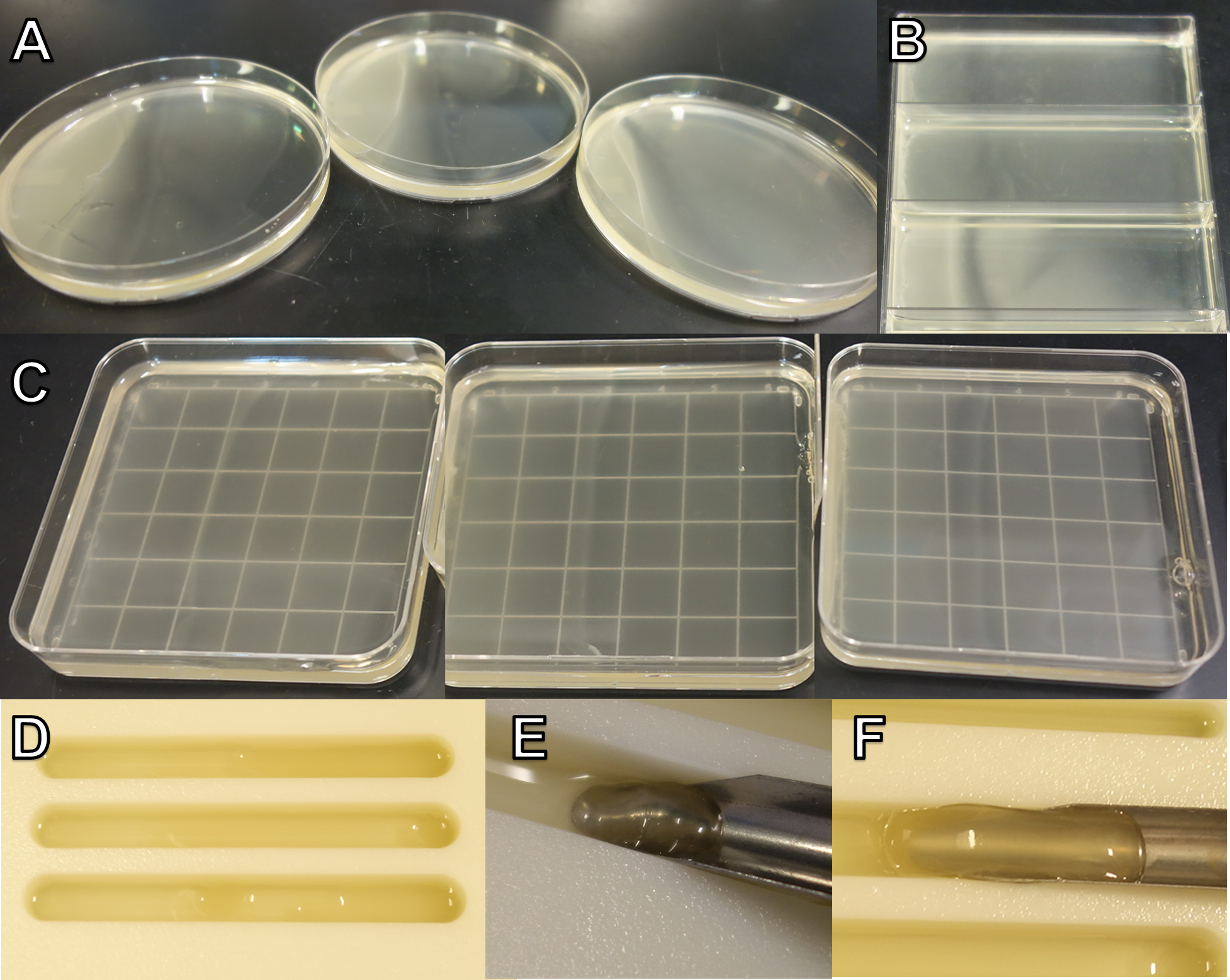
Fig S2.** Soft Agar Gradient Evolution in various growth chambers. 75 ug/mL ciprofloxacin antibiotic gradients were created in A) 100 mm Petri dishes, B) 80 mm culture dishes, C) 100 x 100 mm square Petri dishes, or D) 12.5 x 110 mm polycarbonate channels. Bacteria were then inoculated in a line along the left-most edge of the chamber. After 24 hr of incubation bacteria had traversed each plate, and the chambers were then photographed. E-F) Closeup of the polycarbonate chamber, highlighting E) absence or F) presence of bacterial growth.


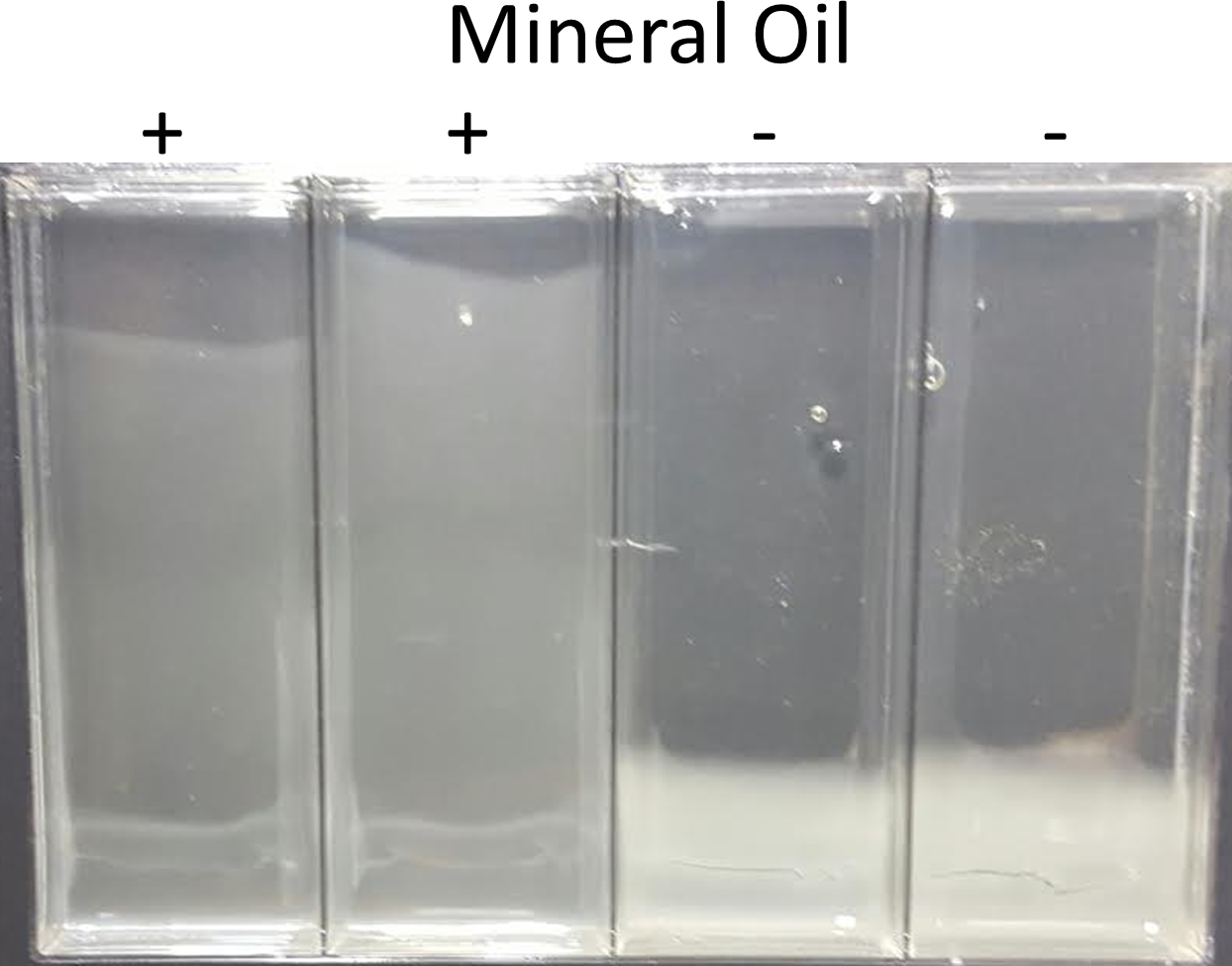


**FIG S3**. Evolution with and without a layer of mineral oil. 50 uL of speed-selected *E. coli* MG1655 was added to lanes containing up to 10 mg/L polymyxin B. 2.5 mL of oil was then added to the two left lanes and the plate was incubated at 37 ℃ for 20 hr. Without a layer of oil lanes show higher cell densities but slower movement across the plate.


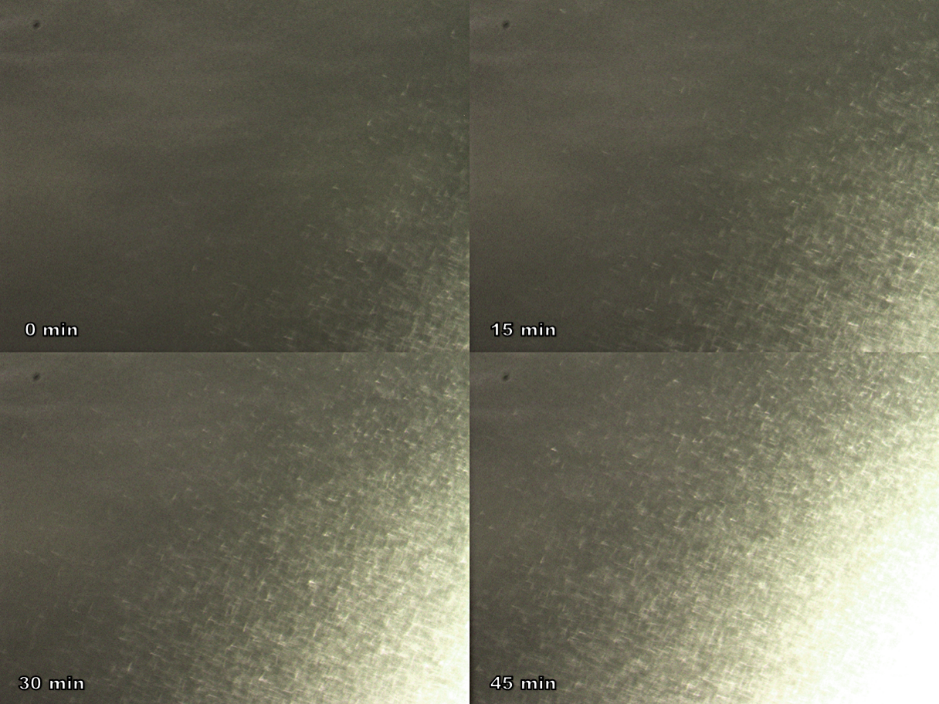


**Fig. S4.** Movement of speed-selected *E. coli* MG1655 in soft agar, 150x magnification. Cells appear as pinpoints of light, emerging from the bottom-right of the plate. Cells at the outer edge move in a random-walk pattern towards the top-left. Movement is unbiased far from the band of high-density cells, preventing rapid dispersal throughout the plate. Cells behind the high-density band of cells are fixed in place.


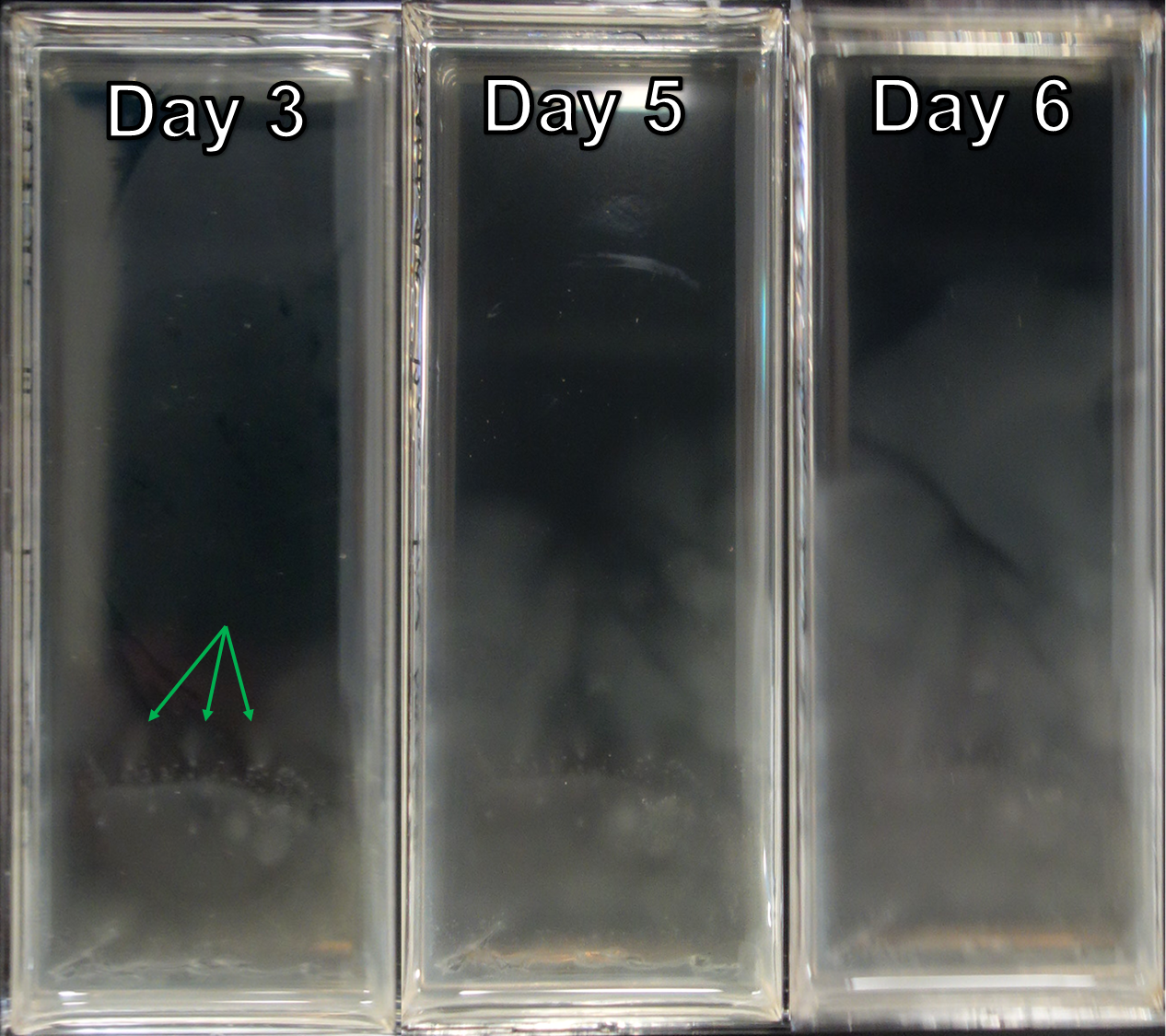


**Fig S5.** Evolution of resistance to trimethoprim. SAGE plates inoculated with speed-selected *E. coli* MG1655 were incubated at 37 ℃ for seven days. Shown here is one representative lane, containing up to 50x the initial MIC of trimethoprim at a slope of 12.5 mm. Mutations visible on day 3 are marked with arrows. These small cones grow to form the bulk of the plate on days 5 and 6, without being appreciably slowed by the increasing concentration of trimethoprim in the upper portion of the plate.
